# Sustained-Release Delivery of Prostacyclin Analogue Enhances Bone Marrow-Cell Recruitment and Yields Functional Benefits for Acute Myocardial Infarction in Mice

**DOI:** 10.1371/journal.pone.0069302

**Published:** 2013-07-19

**Authors:** Yukiko Imanishi, Shigeru Miyagawa, Satsuki Fukushima, Kazuhiko Ishimaru, Nagako Sougawa, Atsuhiro Saito, Yoshiki Sakai, Yoshiki Sawa

**Affiliations:** 1 Department of Cardiovascular Surgery, Graduate School of Medicine, Osaka University, Osaka, Japan; 2 Medical Center for Translational Research, Osaka University Hospital, Osaka, Japan; 3 Research Headquarters, ONO Pharmaceutical CO., LTD., Osaka, Japan; Tokai University, Japan

## Abstract

**Background:**

A prostacyclin analogue, ONO-1301, is reported to upregulate beneficial proteins, including stromal cell derived factor-1 (SDF-1). We hypothesized that the sustained-release delivery of ONO-1301 would enhance SDF-1 expression in the acute myocardial infarction (MI) heart and induce bone marrow cells (BMCs) to home to the myocardium, leading to improved cardiac function in mice.

**Methods and Results:**

ONO-1301 significantly upregulated SDF-1 secretion by fibroblasts. BMC migration was greater to ONO-1301-stimulated than unstimulated conditioned medium. This increase was diminished by treating the BMCs with a CXCR4-neutralizing antibody or CXCR4 antagonist (AMD3100). Atelocollagen sheets containing a sustained-release form of ONO-1301 (n = 33) or ONO-1301-free vehicle (n = 48) were implanted on the left ventricular (LV) anterior wall immediately after permanent left-anterior descending artery occlusion in C57BL6/N mice (male, 8-weeks-old). The SDF-1 expression in the infarct border zone was significantly elevated for 1 month in the ONO-1301-treated group. BMC accumulation in the infarcted hearts, detected by in vivo imaging after intravenous injection of labeled BMCs, was enhanced in the ONO-1301-treated hearts. This increase was inhibited by AMD3100. The accumulated BMCs differentiated into capillary structures. The survival rates and cardiac function were significantly improved in the ONO-1301-treated group (fractional area change 23±1%; n = 22) compared to the vehicle group (19±1%; n = 20; P = 0.004). LV anterior wall thinning, expansion of infarction, and fibrosis were lower in the ONO-1301-treated group.

**Conclusions:**

Sustained-release delivery of ONO-1301 promoted BMC recruitment to the acute MI heart via SDF-1/CXCR4 signaling and restored cardiac performance, suggesting a novel mechanism for ONO-1301-mediated acute-MI heart repair.

## Introduction

Despite a number of medical and interventional treatments have been developed to treat acute myocardial infarction (AMI), the treatment for massive AMI has not been fully established. Myocardial infarction (MI) is a progressive disease, characterized by massive ischemic necrosis of the myocardial tissue and subsequent inflammation. This leads to cardiac remodeling that exacerbates the oxygen shortage in the surviving cardiac tissue. These pathological and functional deteriorations eventually cause end-stage heart failure. To delay the progression of heart failure, it is essential to suppress inflammation and fibrosis and to improve bloodflow supply in the injured myocardium consecutively. Recently, stromal cell-derived factor (SDF)-1 and its corresponding receptor CXCR4 have been shown to play prominent roles in homing of bone marrow cells (BMC) which promotes neovascularization and prevention of apoptosis via paracrine mechanism [1], [2], [3], [4].

ONO-1301 (({5-[2-({[(1E)-phenyl(pyridin-3-yl)methylene]amino}oxy)ethyl]-7,8-dihydronaphthalen-1-yl}oxy)acetic acid) is a synthetic prostacyclin agonist. As it lacks the typical prostanoid structure of a five-membered ring and an allylic alchol, ONO-1301 is chemically and biologically stable *in vivo*. In addition, thromboxane A2 synthethase is inhibited by ONO-1301, resulting in the promotion of endogenous prostacyclin synthesis. ONO-1301 has been reported to induce the production of endogenous hepatocyte growth factor (HGF) and vascular-endothelial growth factor (VEGF) in fibroblasts by stimulating cAMP production [5], [6], [7], [8]. The administration of a slow-release form of ONO-1301 shows therapeutic potential, mainly due to the restoration of bloodflow in MI models of rat and swine and in a cardiomyopathic hamster [6], [7], [8]. The potential mechanism of the functional benefits of ONO-1301 mainly result from the enhanced secretion of growth factors, such as HGF and VEGF, which induce angiogenesis, restore bloodflow, and attenuate the progression of fibrosis. Recently we identified that ONO-1301 also upregulates SDF-1 secretion in the fibroblasts. Enhanced BMC homing in the MI heart by ONO-1301 therapy is attractive therapeutic modality. We thus hypothesized that ONO-1301 can induce BMC accumulation mediated by the upregulation of SDF-1 to elicit functional improvement in a mouse model of MI.

## Methods

This study was carried out in strict accordance with the recommendations in the Guide for the Care and Use of Laboratory Animals of the National Institutes of Health. The protocol was approved by the Committee on the Ethics of Animal Experiments of the Osaka University (H23–123). All surgery was performed under sodium pentobarbital or isoflurane anesthesia, and all efforts were made to minimize suffering.

ONO-1301 and a slow-release form of ONO-1301 were purchased from ONO Pharmaceutical Co. Ltd. (Osaka, Japan) [7], [8], [9].

### Migration Assay

Normal human dermal fibroblasts (NHDFs; Takara bio, Shiga, Japan) were cultured with or without ONO-1301 for 72 hours. The SDF-1 concentration in the culture supernatants was measured by ELISA (R&D systems, MN). BMCs were obtained from a green fluorescent protein (GFP)-transgenic mouse [C57BL/6-Tg(CAG-EGFP); Japan SLC, Inc., Shizuoka, Japan], and their migration toward the supernatants was assessed using a culture insert system (BD Falcon). The number of migrated BMCs was determined using fluorescence microscopy (Carl Zeiss, Göttingen, Germany).

### Mouse AMI Model and Sheet Transplantation

An AMI model was generated by permanent ligation of the left anterior descending artery (LAD) in 10-15-week-old male C57BL/6N, BALB/cA, or BM-GFP chimera mice [10]. ONO-1301 microspheres and control microspheres were resuspended in saline at 10 mg/ml and added to atelocollagen sheets just before transplantation. Five minutes after the LAD ligation, atelocollagen sheets that included ONO-1301-containing microspheres (ONO-1301-treated group, n = 40) or empty microspheres (vehicle group, n = 40) were fixed onto the surface of the anterior left ventricular (LV) wall. The mice were euthanized 7, 21, and 28 days after the LAD ligation and ONO-1301 administration.

### Assessment of BMC Homing

BMCs harvested from BALB/cA mice were labeled by Xenolight DiR (Caliper Life Sciences, MA) following the manufacturer’s instructions and injected into the tail vein of BALB/cA mice after the MI and ONO-1301 treatment. On days 1 and 3, the whole-body imaging of the mice was measured by an *in vivo* imaging system (IVIS, Caliper Life Sciences).

### Assessment of Cardiac Function and Survival

Cardiac function was assessed using an echocardiography system equipped with a 12-MHz transducer (GE Healthcare, WI) 4 weeks after MI and ONO-1301 treatment. The LV areas were measured, and LV fractional area change (FAC) was calculated as (LVEDA-LVESA)/LVEDA×100, where LVEDA and LVESA are the LV end-diastolic and end-systolic area, respectively.[10] The mice were housed in a temperature-controlled incubator for 28 days post-treatment to determine their survival.

### Histological Analysis

Frozen sections (8 µm) of hearts were stained with antibodies against von Willebrand factor (vWF; Dako, Glostrup, Denmark) and CD31 (Abcam, UK). The secondary antibody was Alexa 546 goat anti-rabbit (Life Technologies, CA). Counterstaining was performed with 6-diamidino-2-phenylindole (DAPI; Life Technologies). The sections were also stained with isolectin (Life Technologies) following the manufacturer’s instructions. To count GFP-positive cells, isolectin-positive cells, and CD31-positive capillary densities, 10 images were captured for each specimen. Capture and analysis were performed using Biorevo (Keyence, Japan). To analyze the myocardial collagen accumulation, heart sections were stained with Masson’s trichrome. The collagen volume fraction in the peri-infarct area was calculated.

### Quantitative Real-time PCR

The total RNA was isolated from the peri-infarct area using the RNeasy Mini Kit and reverse transcribed using Omniscript Reverse transcriptase (Qiagen, Hilden, Germany). Quantitative PCR was performed with a PCR System (Life Technologies). The expression of each mRNA was normalized to that of glyceraldehyde-3-phosphate dehydrogenase (GAPDH). The primers and probes are shown in Table S1 in File S1.

### Statistical Analysis

Data are expressed as the mean ± SEM. The data distributions were checked for normality. Comparisons between 2 groups were made using the Student’s *t*-test. For comparisons among 3 or more groups, one-way analysis of variance (ANOVA) followed by Fisher’s protected least significant difference (PLSD) test were used. The survival curves were prepared using the Kaplan-Meier method and compared using the log-rank test. All *P-*values are two-sided, and values of *P*<0.05 were considered to indicate statistical significance. Statistical analyses were performed using the StatView 5.0 Program (Abacus Concepts, Berkeley, CA) and Statcel2 (The Publisher OMS Ltd., Saitama, Japan).

An expanded Methods section can be found in the online-only in File S1.

## Results

### ONO-1301 Enhanced BMC Migration via SDF-1/CXCR4 Signaling

**Figure 1:**
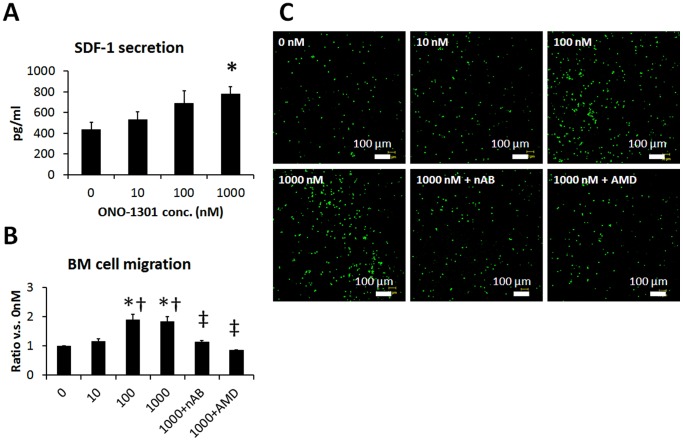
ONO-1301 enhanced SDF-1 secretion and BMC migration via SDF-1/CXCR4 signaling *in vitro*. NHDFs were stimulated with ONO-1301 for 72 hours, then the SDF-1 concentration in the culture medium was determined by ELISA (n = 3 each, *P<0.05 vs. 0 nM). A) Number of BMCs that migrated toward the conditioned medium from ONO-1301-stimulated-NHDFs (0, 10, 100, or 1000 nM ONO-1301, n = 6; 1000 nM+nAB or 1000 nM+AMD, n = 3). *P<0.05 vs. 0 nM, †*P*<0.05 vs. 10 nM, ‡*P*<0.05 vs. 1000 nM, §*P*<0.05 vs. SDF-1. nAB, CXCR4-neutralizing antibody; AMD, CXCR4 antagonist AMD3100. B) Representative pictures of BMCs that had migrated to the medium from ONO-1301-stimulated BMCs. Green, BMCs.

The effect of ONO-1301 on the SDF-1 secretion by NHDFs was evaluated by ELISA. As shown in Fig. 1A, the SDF-1 concentration in the NHDF culture supernatants increased in an ONO-1301 concentration-dependent manner. The SDF-1 concentration in the culture supernatant of 1000 nM ONO-1301-treated cells was significantly greater than that of cells cultured in the absence of ONO-1301 (Fig. 1A). To investigate the BMC migration toward ONO-1301-treated NHDF conditioned medium, a migration assay was performed using a modified Boyden chamber with 8-µm pores. The number of migrated BMCs was significantly greater in the conditioned medium of cells treated with 100 and 1000 nM ONO-1301 compared to that of cells treated with 0 and 10 nM ONO-1301. The BMC migration to the 1000 nM ONO-1301 conditioned medium was diminished by treating the BMCs with a CXCR4-neutralizing antibody or CXCR4 antagonist (AMD3100) (Fig. 1B, C).

### SDF-1-mediated BMC Accumulation in the ONO-1301-treated Infarcted Hearts

**Figure 2:**
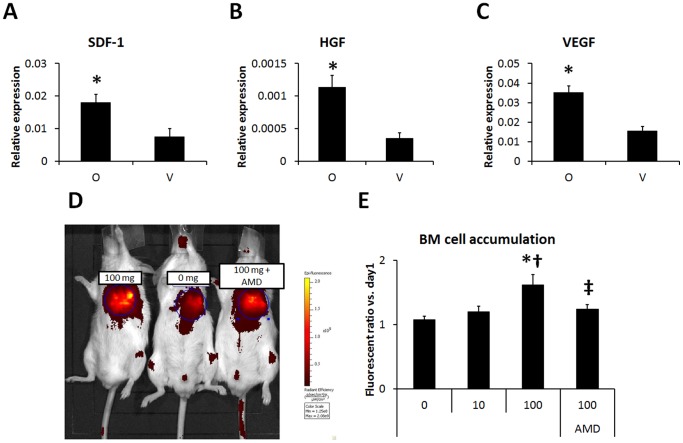
ONO-1301 enhanced SDF-1 secretion and BMC migration via SDF-1/CXCR4 signaling after MI. A–C) The SDF-1, HGF, and VEGF expression at the border zone of the infarcted area was measured by quantitative RT-PCR. The expression levels of these cytokines were higher in the ONO-1301-treated (O) group compared to the vehicle (V) group. (O group, n = 7; V group, n = 7–8; *P<0.05 vs. V group). The expression relative to GAPDH is shown. D) BMC migration to ONO-1301-treated infarcted myocardium was evaluated using IVIS. Representative picture of IVIS at day 3. Left: 100 mg/Kg, Center: 0 mg/Kg, Right: 100 mg/Kg+AMD3100 (AMD). E) The number of accumulated BMCs was greater in the 100 mg/kg ONO-1301-treated infarcted heart compared to the 0 and 10 mg/kg ONO-1301-treated infarcted heart. When BMCs treated with AMD were injected, the BMC accumulation decreased in the 100 mg/Kg ONO-1301-treated infarcted heart compared with the untreated-BMC-injected heart (0 mg/Kg, n = 4; 10 mg/Kg, n = 8; 100 mg/Kg, n = 5; 100 mg/Kg+AMD3100, n = 4; *P<0.05 vs. 0 mg/Kg, †*P*<0.05 vs. 10 mg/Kg, ‡*P*<0.05 vs. 100 mg/Kg).

The effect of ONO-1301 on SDF-1 expression in the infarcted hearts was evaluated by quantitative RT-PCR. Twenty-eight days after treatment, the SDF-1 expression in the border area of the ONO-1301-treated heart was significantly greater than that in the vehicle-treated heart (Fig. 2A). The HGF and VEGF expressions were also increased by ONO-1301 treatment (Fig. 2B, C). After LAD occlusion, ONO-1301 treatment, and intravenous injection of labeled BMCs, the BMC accumulation in the infarcted heart was evaluated by an in vivo imaging system. The proportion of BMCs in the heart showed a trend toward upregulation, dependent on the dose of ONO-1301 (Fig. 2D). Hearts treated with 100 mg ONO-1301/kg body weight showed significantly more accumulated BMCs than those treated with 0 or 10 mg ONO-1301. In 100 mg/kg ONO-1301-treated hearts, CXCR4 antagonization significantly decreased the BMC accumulation (Fig. 2D). To identify the recruited BMCs *in vivo*, the acute MI model was prepared using chimera mice by transplanting GFP-expressing bone marrow into irradiated C57BL/6 mice. The BMCs of the C57BL/6 transplant recipients were largely replaced by GFP-expressing BMCs (91.8+/−4.3%, figure S1 in File S1). The single-organ analyses using GFP-BM chimera mouse at day 7 also showed increased BMC accumulation in the ONO-1301-treated myocardium (figure S2 in File S1).

### Differentiation of BMCs in the Infarcted Myocardium

**Figure 3:**
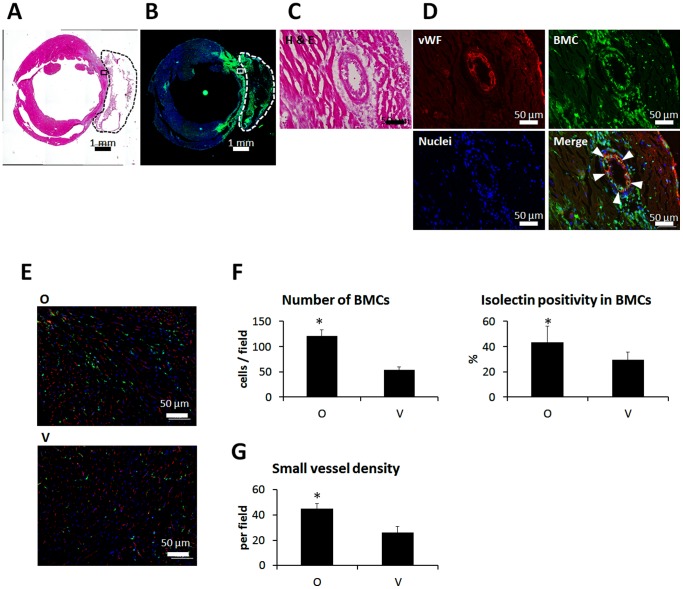
BMCs differentiated into capillary structures in the infarcted area after MI and ONO-1301 treatment. Representative macro image of H and E staining seven days after MI and ONO-1301 treatment. The transplanted sheet is enclosed by a dashed line. A) Serial section of A. The BMCs displayed GFP. B) High-magnification image of the boxed region in A. C) Serial section of C. Arrowheads indicate vWF-expressing BMCs. Red indicates vWF; green, BMCs; and blue, nuclei. D) Representative images of isolectin-stained BMCs seven days after MI and ONO-1301 treatment. E) BMC accumulation and percentages of isolectin-positive BMCs. The number of BMCs that accumulated in the infarcted myocardium was greater in the ONO-1301-treated (O) group than in the vehicle (V) group. The percentage of isolectin-positive BMCs was also greater in the O group than in the V group. *P<0.05 vs. V group. F) Small vessel density. Small vessels were detected by CD31 immunostaining. The density of small vessels in the O group was greater than in the V group. *P<0.05 vs. V group.

Seven days after MI and ONO-1301 administration to BM-GFP chimera mouse, BMCs were dramatically accumulated in both the infarcted area and the atelocollagen sheet (Fig. 3A, B). Some of the BMCs formed tube-like structures and displayed von Willebrand factor expression (Fig. 3C, D). Isolectin staining showed that a greater percentage of isolectin-positive BMCs accumulated in the myocardium in the ONO-1301-treated (O) group than in the vehicle (V) group (Fig. 3E, F). We also evaluated small blood vessels by CD31 immunostaining. The density of small vessels was greater in the O group than in the V group (Fig. 3G). Immunohistochemical analysis of Connexin 43 and smooth muscle actin, cardiac-lineage and cardiac fibroblast markers, respectively, was also conducted at 3 months, but no co-expression of GFP with either of these markers was observed (figure S3 in File S1).

### Therapeutic Effects of ONO-1301 Administration on Cardiac Performance, Survival, and LV-remodeling at 4 Weeks Post-MI

**Figure 4:**
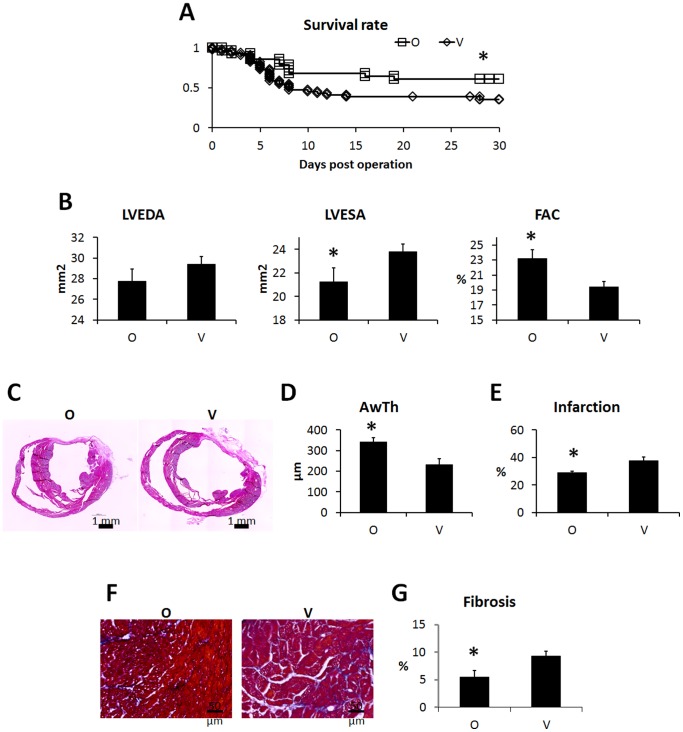
ONO-1301 treatment improved the cardiac performance and survival rate after MI. Survival rates after treatment. The ONO-1301-treated (O) group (n = 33) showed significantly better survival than the vehicle (V) group (n = 48). *P<0.05 vs. V group. A) Evaluation of cardiac performance 4 weeks after treatment. In the O group, the LVESA was smaller, and the FAC was significantly higher compared to the V group (O group, n = 22; V group, n = 20; *P<0.05 vs. V group). B) Representative macro images from each group. C) Quantification of anterior wall thickness. Anterior wall thickness was significantly thicker in the O group (n = 6) compared to the V group (n = 4). *P<0.05 vs. V group. D) Quantification of percent infarction. Infarction was significantly smaller in the O group (n = 6) compared to the V group (n = 4). *P<0.05 vs. V group. E) Representative Masson trichrome staining images at the border zone. F) Quantification of fibrosis. Fibrosis at the border zone was significantly smaller in the O group (n = 6) compared to the V group (n = 4). *P<0.05 vs. V group.

ONO-1301 was detected in the plasma of blood samples from the ONO-1301-treated group 3 weeks after treatment (figure S4 in File S1). The cardiac functions in the MI mice with and without following ONO-1301 treatment were evaluated. Mortality was substantial until 14 days post-LAD ligation in the vehicle group, and similar mortality levels were observed with non-treated MI mice [11]. In contrast, in the ONO-1301-treated group, there was little mortality 7 days after MI, and thus a difference in survival (Fig. 4A). Cardiac performance was evaluated by 2D echocardiography 4 weeks after implantation. The LVEDA was smaller in the ONO-1301-treated group than in the vehicle group, but the difference was not significant. In contrast, the LVESA was significantly smaller, and the LVFAC was significantly greater, in the ONO-1301-treated group than in the vehicle group (Fig. 4B). In the histological analysis, the vehicle group showed a typical MI with a large anterior LV scar and dilatation of the LV cavity. By comparison, the LV of the ONO-1301-treated group was less dilated, and the anterior wall was thicker (Fig. 4C, D). The infarcted area and percent fibrosis were significantly smaller in the ONO-1301-treated than in the vehicle-treated group (Fig. 4C, E–G).

## Discussion

Here, we showed that ONO-1301 promotes BMC accumulation in the injured myocardium. *In vitro*, ONO-1301 enhanced SDF-1 expression, and BMC migration was greater to conditioned medium obtained from ONO-1301-stimulated cells. The enhanced migration was diminished by blocking SDF-1/CXCR4 signaling. Consistent with the *in vitro* experiments, ONO-1301 enhanced the SDF-1 expression of myocardial tissue. High ONO-1301 accelerated the BMC accumulation after MI in a SDF-1/CXCR4-dependent manner. Some BMCs in the infarcted myocardium differentiated into capillary structures within 7 days. Furthermore, the sustained-release delivery of ONO-1301 in the infarcted myocardium also led to functional improvements following MI. Our data suggest that ONO-1301 is a novel inducer of BMC recruitment, and that ONO-1301 treatment may be a promising therapeutic strategy for the clinical treatment of MI.

It is difficult to understand the whole mechanism underlying the functional improvements induced by ONO-1301. It was already reported that ONO-1301 enhances the expression of angiogenic factors HGF and VEGF, leading to angiogenesis and the suppression of fibrosis progression [7], [8], [9]. In this study, we discovered an alternative mechanism for ONO-1301’s therapeutic efficacy in the acute MI mouse, in which the upregulation of SDF-1 promotes BMC accumulation. Stem-cell recruitment and homing are regulated by the interplay of cytokines, chemokines, and proteases. In particular, the SDF-1/CXCR4 axis is central for the mobilization of stem cells from the bone marrow and their homing to ischemic tissues [12]. In the case of ischemic insult, SDF-1 is released by the injured tissue and stimulates the mobilization of progenitor cells from the bone marrow [1], [13]. Furthermore, prostaglandins have been reported to facilitate BMC mobilization via upregulation of CXCR4 expression [14], [15]. In our experimental setting, ONO-1301 was detected from peripheral blood samples 3 weeks after treatment (Fig. S4 in File S1), suggesting that ONO-1301 may similarly act on the bone marrow to promote the BMC mobilization. Thus, BMC recruitment in the injured myocardium may be enhanced by the upregulation of SDF-1 in cardiac fibroblasts and by the direct upregulation of CXCR4 in BMCs located in the bone marrow. In addition, recent reports show the possibility of endogenous regeneration in the injured heart, including proliferation of postnatal cardiomyocytes and cardiac stem cells [16], [17], [18], [19]. While we were unable to detect newly-generated cardiomyocytes derived from BMCs in this study, it would be interesting to evaluate the possibility of cardiomyogenesis involving other cell types.

We observed massive BMC accumulation 7 days after MI, including in the infarcted ventricular wall, where they provided structural support in place of the necrotic cardiomyocytes. The BMCs recruited into the infarcted myocardium may contain various kinds of somatic stem cells, such as endothelial progenitor cells [20], bone marrow-derived stem cells [21], and bone marrow mononuclear cells [2], which have potent therapeutic effects in heart failure [22]. Furthermore, bone marrow-derived mesenchymal stem cells secrete prostaglandin [23], which may act like ONO-1301 and amplify the effects of the ONO-1301-mediated therapy. Kawabe et al. clearly showed that prostaglandin facilitates the recruitment of endothelial progenitor cells [24]. Although further analysis is needed, the enhanced accumulation of BMCs may predispose the damaged heart tissue to better restoration following MI.

Many reports have shown that granulocyte colony-stimulating factor (G-CSF) and granulocyte-macrophage colony-stimulating factor (GM-CSF) also induce BMC mobilization, with therapeutic effects in animal models [25]. However, G-CSF therapy in unselected patients with acute MI did not lead to functional improvements beyond those achieved with conventional therapy. In addition, the administration of GM-CSF in cancer patients has been shown to transiently increase the LV end-systolic dimensions and decrease cardiac contractility [25], [26]. The lack of efficacy of G-CSF therapy in clinical trials may be due, at least in part, to its poor initiation and duration; such therapies are likely to be most beneficial during the early phase after acute MI. Although conventional prostacyclin and its analogs are chemically and biologically unstable, ONO-1301 is a long-acting prostacyclin agonist that exerts stable effects *in vivo*, because it lacks a prostanoid structure. Furthermore, we used a slow-release form of ONO-1301, made by polymerizing it with poly-lactic and glycolic acid; this ONO-1301 could still be detected in the blood 3 weeks after its administration (figure S4 in File S1).

Furthermore, in our *in vitro* analysis, although we used normal human dermal fibroblasts to examine the SDF-1/CXCR-4-dependent BMC migration, the reactivity to ONO-1301 stimulation will differ depending on the cell type. For example, the G-CSF expression was upregulated in some kinds of cells (unpublished data). Thus, together with the upregulation of multiple beneficial cytokines such as HGF and VEGF, because of the longer duration of its activity, ONO-1301 may be more potent than conventional protein-based therapies.

Our data showed that ONO-1301 treatment was a potent inducer of BMC homing. Of the BMCs that accumulated in the infarcted myocardium, 43 percent expressed isolectin, an endothelial cell marker, but the other BMCs had a fibroblastic morphology, and did not express cardiac-lineage or cardiofibroblast markers (Figure S3 in File S1). ONO-1301 administration resulted in the attenuation of cardiac dysfunction, with enhanced BMC accumulation. Further study is required to elucidate the mechanism, but we speculate that paracrine effects of factors released by the BMCs play pivotal roles in the therapeutic efficacy, rather than the transdifferentiation of the BMCs into the cardiac or vascular lineage. The effect of cardioprotective and angiogenic factors secreted by the accumulated BMCs and the direct stimulation of ONO-1301 itself may synergistically increase the angiogenesis and cardioprotection, leading to improved therapeutic results.

In summary, ONO-1301 may be a powerful, long-acting activator of multiple cytokines. In particular, SDF-1 may enhance the BMC accumulation in a SDF-1/CXCR4-signaling-dependent manner, leading to an attenuation of the cardiac dysfunction following MI. Our findings suggest that the method involving a sustained release of ONO-1301 may be adapted as a novel drug delivery system for treating heart failure.

## Supporting Information

File S1 (DOCX)
