# Supplementary material for: Sustained-Release Delivery of Prostacyclin Analogue Enhances Bone Marrow-Cell Recruitment and Yields Functional Benefits for Acute Myocardial Infarction in Mice
Source: PLoS One. 2013 Jul 19;8(7):e69302. doi: 10.1371/journal.pone.0069302 (PMC3716598; doi:10.1371/journal.pone.0069302)

# **Supporting Materials and Methods**

## **Enzyme-Linked Immunosorbent Assay**

To analyze the secreted factors, enzyme-linked immunosorbent assays (ELISAs) were performed. The collected culture supernatants of NHDFs incubated with 0 to 1000 nM ONO-1301 were centrifuged to remove debris and contaminating cells. The SDF-1 content in the undiluted culture supernatants was determined with an ELISA kit (R&D Systems, MN, USA) according to the manufacturer’s instructions (0, 10, 100, or 1000 nM ONO-1301, n=6 each; 1000 nM + neutralizing antibody, 1000 nM + AMD3100, n=3 each).

## **Migration assay**

Normal human dermal fibroblasts (NHDFs) were cultured in Dulbecco’s modified Eagle’s medium (DMEM) (Nacalai Tesque, INC., Kyoto, Japan) containing 10% fetal bovine serum. The cells were serum-starved overnight, then cultured with or without ONO-1301 for 72 hours. The culture supernatants were collected, and the SDF-1 concentration was determined using an ELISA kit (R&D Systems, MN). Each conditioned medium was then concentrated twofold using Amicon Ultra centrifugal filters (Millipore, Billerica, MA).

Whole BMCs were harvested by flushing the femurs of a GFP mouse (Japan SLC, Inc., Shizuoka, Japan) with phosphate-buffered saline (PBS). The red blood cells were lysed with ACK buffer (150 mM NH_4_Cl, 10 mM KHCO_3_, 0.1 mM EDTA, pH 7.2) at 4˚C for 10 min. The cells were washed three times with PBS and resuspended in 0.5 mL of PBS. The FluoroBlock 24-Multiwell Insert System (BD Falcon, NJ) containing 8-μm pore size filters was used for the migration assay. A suspension of 50,000 BMCs in DMEM (350 µL) containing 10% FBS was applied to the upper chamber. In some experiments, the BMCs were incubated for 30 minutes at 37°C with 5 μg/mL AMD3100 or 10 μg/mL CXCR4 neutralizing antibody (R&D Systems). The lower chamber was filled with 1.0 mL of concentrated culture supernatant with or without 5 μg/mL AMD3100 and 10 μg/mL CXCR4 neutralizing antibody (R&D Systems). After incubation at 37°C for 2 hours, the number of migrated cells was counted in five randomly chosen fields under 100× magnification under a fluorescence microscope, LSM 510 META (Carl Zeiss, Göttingen, Germany).

## **Animal experiments**

The mouse myocardial infarction (MI) model was created by left anterior descending artery (LAD) ligation as described previously. Mice (10-15-weeks old, male C57BL/6J, Japan Clea, Tokyo Japan) were anesthetized by inhalation of isoflurane (1.5%, 1L/min, Mylan Inc., Pittsburgh, PA) provided by an anesthetic gas machine (DS Pharma, Osaka, Japan). The anesthetized mice were intubated in an endotracheal manner, and positive pressure ventilation was maintained with a ventilator (room air, 90 cycles/minute, tidal volume 1 ml, Shinano, Tokyo, Japan). The heart was then exposed through a left lateral thoracotomy. With minimal manipulation of the fat pad surrounding the heart, the LAD could easily be visualized. The LAD was ligated with an 8-0 prolene suture (Johnson & Johnson, NJ, USA) at 1 mm distal to the left atrial appendage, immediately after bifurcation of the major left coronary artery. The myocardial ischemic area was visually assessed to confirm that the LAD ligation had consistent ischemic effects. Procedure-related mortality, which occurred prior to chest closure, was consistently 6% in all the experimental groups, suggesting that the level of acute myocardial ischemia was constant. Within 5 minutes after LAD ligation, the mice were randomly allocated into 2 groups: those that underwent transplantation of atelocollagen sheets (Koken Co., Ltd., Tokyo, Japan) that included 10 mg/kg ONO-1301-microspheres (O group; n=33) and microspheres only (V group; n=48). ONO-1301-microspheres or empty microspheres which were freshly suspended in saline were dropped onto 3-atelocollagen-sheets (diameter: 6 mm) and used within 3 hours. The pericardium was closed to prevent dislocation of the atelocollagen sheets. The mice were allowed to recover under care.

The mice were euthanized 7, 21, and 28 days after surgery by intravenous injection of pentobarbital (200 mg/kg body weight; DS Pharma) and 30 mM potassium chloride (Wako Pure Chemical Industries, Osaka, Japan) to cause cardiac arrest in diastole under terminal anesthesia, and the heart was excised.

On day 28, the specimens for RNA analysis were dissected. The right ventricular free wall, and three regions representing the infarction, peri-infarction, and remote areas were removed and soaked in RNA Later (Qiagen, Hilden, Germany, O, n=9; V, n=10). The specimens for H and E staining, Masson’s trichrome staining, and von Willebrand factor staining were cut into 4 segments, embedded in OCT compound (Sakura Finetek Japan, Tokyo, Japan), and snap frozen in liquid nitrogen (O, n=6; V, n=4). These mice were also used for survival-rate analysis (O, n=33; V, n=48), but cases of accidental death were excluded.

To detect the BMC accumulation level, MI was created in mice (8-week-old,BALB/cA, purchased from Japan Clea) by the same method described above. The mice received transplants of atelocollagen sheets containing 0, 10, or 100 mg/kg ONO-1301. Four to five hours after LAD ligation, BMCs harvested from BALB/cA mice were injected into the tail vein (5×10^6^/mouse). These BMCs had been stained with Xenolight DiR (Caliper Life Science, MA, USA) and treated with or without 5 μg/ml AMD3100 (Sigma-Aldrich, MO) for 30 min at 37℃ (0 nM, n=4; 10 nM, n=7; 100 nM, n=5, 100 nM + AMD, n=4). One and 3 days after MI and ONO-1301 treatment, the accumulated cell dose of ONO-1301 was measured using IVIS (Caliper Life Sciences, MA).

## ***Generation of BM GFP-chimera mice***

BMCs were prepared from C57BL/6 transgenic mice that ubiquitously expressed enhanced green ﬂuorescent protein (EGFP; Japan SLC). The recipient mice (C57BL/6, 8-wks-old, male, Japan SLC) were exposed to 10 Gy of X-irradiation prior to receiving 5×10^6^ BMCs. To verify the reconstitution of bone marrow after transplantation by this protocol, flow cytometry analysis was conducted 6 weeks after the BM transplantation, and showed that the peripheral blood cells consisted of 90% GFP-positive cells (Supplemental figure 1).

## **Quantitative real time PCR**

The total RNA was isolated from stored specimens using the RNeasy Mini Kit (Qiagen) and reverse transcribed with Omniscript Reverse transcriptase (Qiagen). Quantitative PCR was performed with the ABI 7500 Fast Real-Time PCR System (Life Technologies) using Taqman Universal Master Mix (Life Technologies). The mRNA copy number was measured in triplicate. The primers and probes are shown in the Table. All probes were designed with a 5’ fluorogenic probe 6FAM and a 3’ quencher TAMURA. The results were normalized to glyceraldehyde-3-phosphate dehydrogenase (GAPDH).

## **Histological analysis**

Histological analyses of the hearts were performed 7 and 28 days after transplantation. The hearts were cut into 8-μm sections. The sections were stained with antibodies for von Willebrand factor (vWF; 1:500 dilution; Dako, Glostrup, Denmark), Smooth muscle actin (SMA; 1:200, Abcam, Cambridge, UK), and Connexin 43 (Cx43; 1:1000 dilution; Sigma-Aldrich, MO, USA). The secondary antibody was Alexa 546 goat anti-rabbit (1 μg/ml; Life Technologies, CA, USA). Counterstaining was performed with 6-diamidino-2-phenylindole (DAPI; 1 μg/ml; Life Technologies). Images were captured by fluorescence microscopy (Keyence, Osaka, Japan). Routine hematoxylin-eosin staining and Masson’s trichrome staining were performed to analyze the collagen accumulation. The infarct size was calculated as the percentage of the scar distance for each LV circumference. The collagen volume fraction in the peri-infarct area was calculated as the percentage of the myocardium. The data were collected from 10 individual views per heart at a magniﬁcation of ×200. BZ-9000 (Keyence) and MetaMorph (Molecular Devices, CA, USA) software was used for quantitative morphometric analyses.

## **Myocardial echocardiography**

Echocardiography examinations were performed 4 weeks after cell transplantation by an investigator blinded to the group identities (O, n=22; V, n=20)). Two-dimensional, targeted M-mode tracings were obtained at the level of the papillary muscles with an echocardiography system equipped with a 12-MHz transducer (GE Healthcare, WI, USA). The left ventricular (LV) dimensions were measured following the method of the American Society of Echocardiology from at least 3 consecutive cardiac cycles. Three readings were obtained from each mouse and averaged. The LV fractional area shortening (FAC) was calculated as (LVEDA-LVESA)/LVEDA×100, where LVEDA is the LV end-diastolic area and LVESA is the LV end-systolic area.

## **Evaluation of survival rate after the operation**

To evaluate the life-saving effect of the implantation, survival rates were determined. The mice were housed for 28 days after the operation, and the survival rates of the mice in the O and V groups were evaluated by the Kaplan–Meier method. The pairwise comparison was performed by a log-rank test.

# **Table S1. Primers and probes used in this study.**

|  | Forward primer | Reverse Primer | Probe | Accession number |
| --- | --- | --- | --- | --- |
| GAPDH | GCATGGCCTTCCGTGTTC | GATGCCTGCTTCACCACCTT | CCGCCTGGAGAAACCTGCCAAGTATG | M32599 |
| HGF | AGGTCCCATGGATCACACAGA | GCCCTTGTCGGGATATCTTTCT | ACCAGCAGACACCACACCGGCA | BC119228 |
| SDF-1 | AGCTGTGCTCAGAGACCCTTTC | ACAGAGGTGAGAAGCGGAAGTC | TAGCCGTTCCTGCTCTCTGCTTGCC | BC00664 |
| VEGF | GCACTGGACCCTGGCTTTACT | TCATGGGACTTCTGCTCTCCTT | ACCATGCCAAGTGGTCCCAGGCT | M95200 |

# **Supporting figure legends**

## **Supporting figure 1. BM-GFP chimerism.** Eight weeks after radiation and transplantation of GFP-expressing bone marrow, the GFP positivity of the peripheral blood cells was checked using flow cytometry, and found to be 91.8 +/- 4.3 % (n=16). The X-axis shows GFP intensity. Green line shows the BM-GFP chimera mouse-derived peripheral blood sample. Filled curve shows the wild-type mouse-derived peripheral blood sample.

## **Supporting figure 2. BMC accumulation after ONO-1301 treatment.** Seven days after treatment, higher levels of BMC accumulation were detected in ONO-1301-treated MI hearts compared to control (Vehicle) hearts. There were no significant differences between the O and V groups in the liver, spleen, and lung. In the kidney, the signal intensity in the O group was significantly higher than in the V group.

## **Supporting figure 3. Immunostaining of the ONO-1301-treated BM-GFP chimera mouse heart.** Three months after LAD ligation and ONO-1301 treatment, BMCs were detected in the scar area. GFP-positive BMCs were negative for SMA and Cx43. Bar=50 μm

## **Supporting figure 4. ONO-1301 concentration in plasma.** Three weeks after treatment, ONO-1301 was detected in the plasma of blood samples in the ONO-1301-treated (O) group (2.9 +/- 1.5 ng/ml, n=3).

## Figure S1


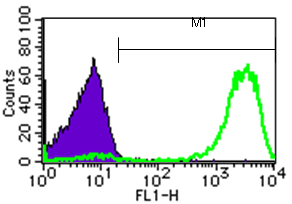


## Figure S2


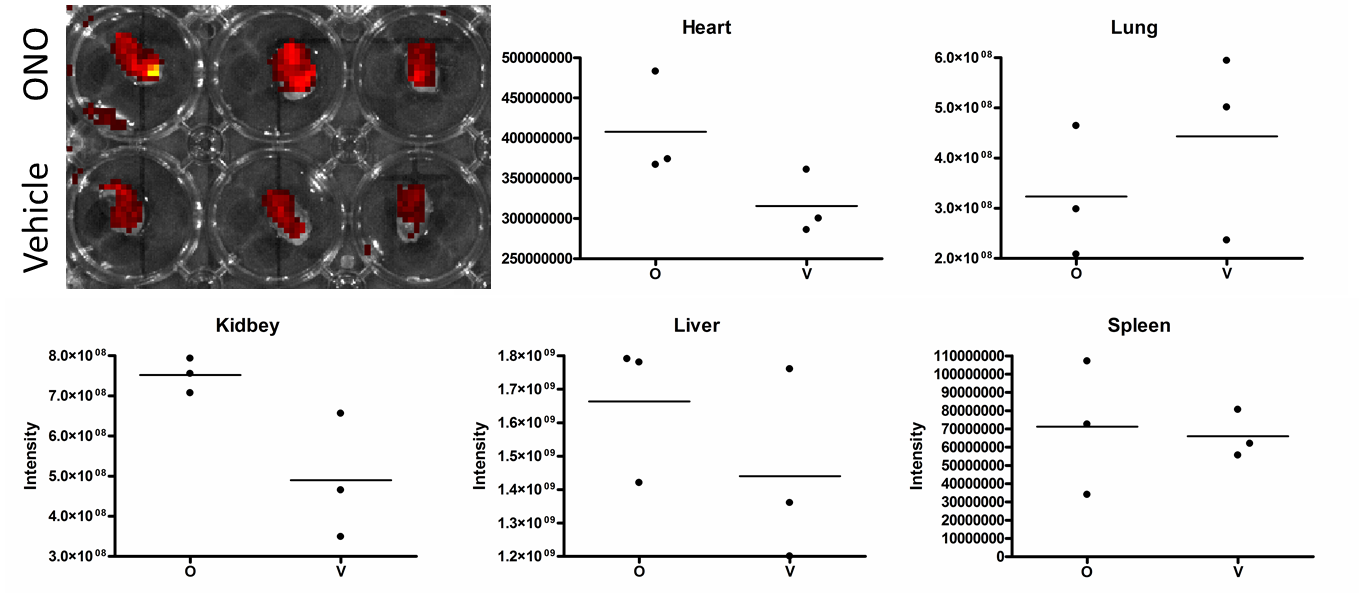


## Figure S3


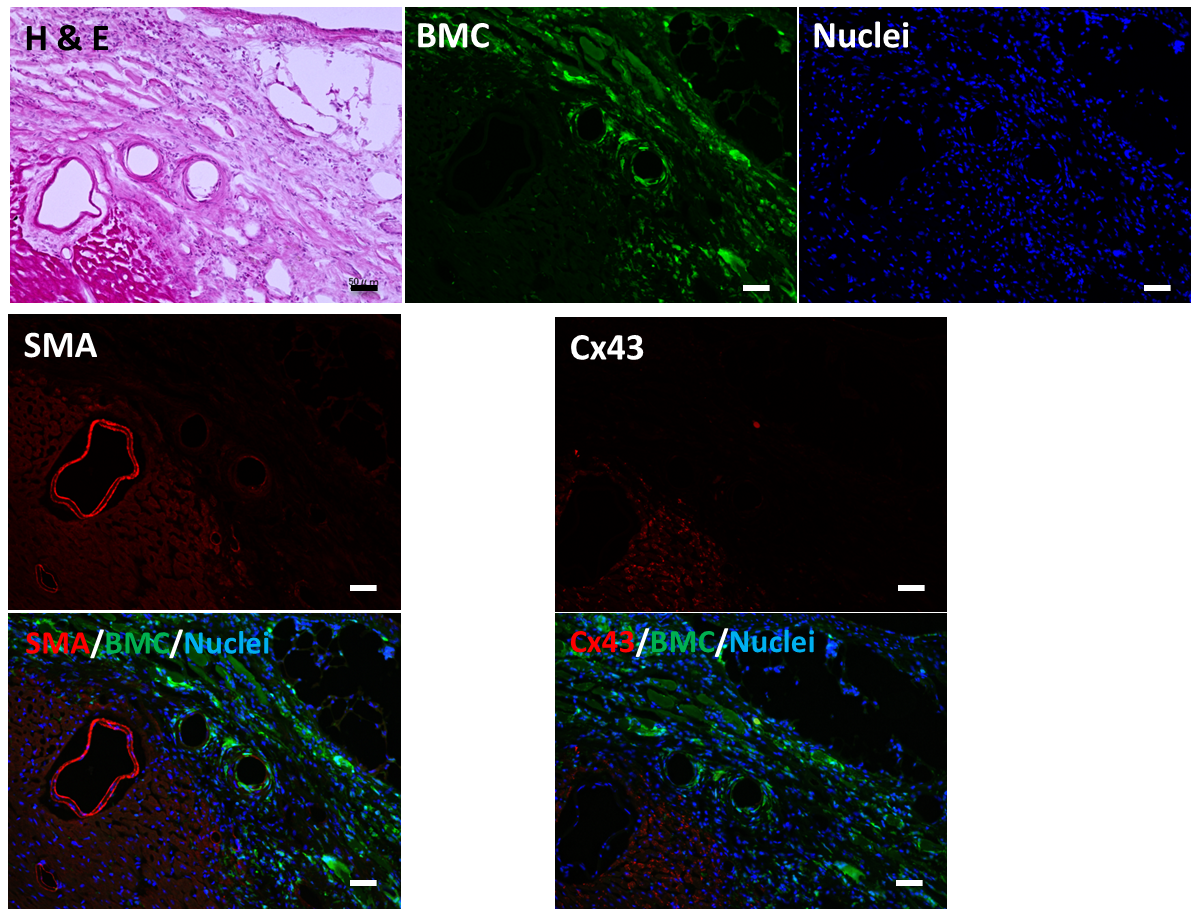


## Figure S4


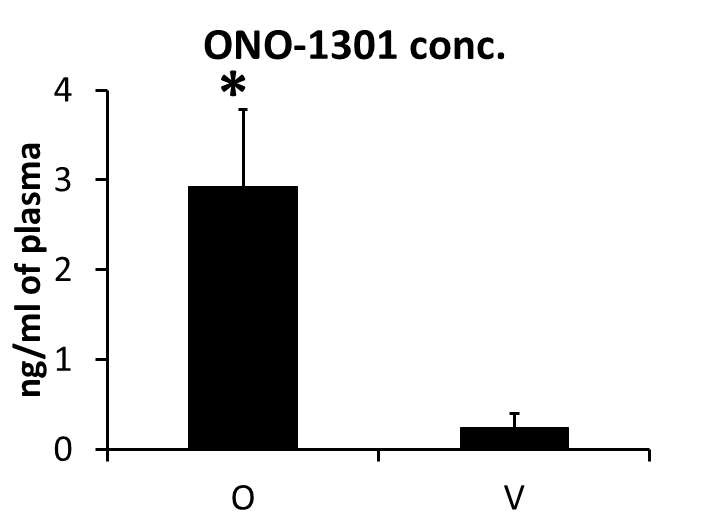

Supplement: File S1 — (DOCX) [file pone.0069302.s001.docx]
